# Hospital Meetings

**Published:** 1902-01-25

**Authors:** 


### HOSPITAL MEETINGS.

### ITALIAN HOSPITAL.

THE annual meeting of governors of the Italian Hospital was held on Saturday last (18th inst.), his Excellency the Italian Ambassador (Cav. Alberto Pansa) presiding.

The report of the committee of management stated that the first complete 12 months' work in the hospital's new home had been a period showing marked increase of good effected among the sick and injured poor. This increase, though gratifying and proving the need and value of the hospital, had, of necessity, added very considerably to the ordinary expenditure; while, owing to the many recent demands upon the charitable public, the income had, like that of many other institutions, come far short of what was absolutely needful. The in-patients treated numbered 520, and the out-patients 8,103—together 8,623. As showing the cosmopolitan character of the work, it was pointed out that of this total 3,630 were Italians, 4,199 were British, and 794 various other nationalities. A further increase in the number of availabls beds had been made, and of the 50 for which the hospital was designed only 10 now remained closed for lack of funds. The ordinary expenditure for the year was £2,256, the ordinary income only £1,599, showing a deficit of £657. Annual subscriptions amounted to £415, as compared with £371 in 1900; but the total of donations, £608, showed a falling-off of £321. A special collection for the endowment of a bed in memory of his late Majesty King Humbert undertaken by Com. L. Allatini (Consul-General) and Cav. Luciano de Rin, was in progress, and had so far resulted in a total of nearly £800. In the closing weeks of the year the committee received the intelligence, through the hospital's bankers, that an anonymous friend had promised £500 "in grateful recognition of Italy's friendship for England, especially during the present war," toward the total of £2,500 required to open the remaining beds and make good the deficit in income if the balance were collected by the end of January, 1902. This period had since been extended to March 31st, provided such progress with the collection could be shown from time to time as would warrant the hope that the balance would be collected by that date.

The committee expressed gratification at being able to report a continuance of the honour conferred on the hospital by the Royal House of Savoy, their Majesties the King and Queen of Italy having consented to become patrons in succession to his late Majesty King Humbert. The letter received was as follows, addressed from Rome to the President of the Committee of Management:—

"Their Majesties the King and Queen have graciously assented to the wish expressed by the Committee of the Italian Hospital in London to honour it with their high patronage.

"Their Majesties, in giving their patronage to this benevolent institution, mean to testify the great interest and special consideration they have for a work which merits the greatest praise and is so advantageous to the poor of the Italian colony living in the English Capital; and express their wish that, with the goodwill of all interested in it, it may always fulfil the noble and patriotic purpose for which it was founded.

"With their Majesties' thanks for the respectful homage that prompted the Committee to ask for the Royal patronage,

I beg to remain,

E. PONZIO-VAGLIA,

*Minister.*

The committee also reported the acceptance of the presidency of the hospital by the Italian Ambassador, in succession to the late Baron de Renzis; and that the vice-presidency had been accepted by Lord Rosebery. Sir Samuel Montagu had undertaken the office of honorary treasurer. During the year the committee elected the following new members to the committee of management to complete the number authorised by the rules, viz., Lady Mary Howard, Miss Bianconi, the Duke of Newcastle, Lord Edmund Talbot, M.P., the Right Hon. Geo. Wyndham, M.P., Maj.-Gen. John Ramsay Slade, C.B., Mr. M. Grancini, and Mr. Joseph Sperati.

A warm tribute was paid to the late Sir William MacCormac, by whose death the hospital had lost one who from the charity's foundation had ever been among its truest friends. Both as a member of the committee of management and professionally as consulting surgeon, he was ever ready to devote his brilliant attainments and his time to secure the hospital's good, and in lamenting his death the committee desired to place on record their grateful acknowledgment of his devoted service and friendship to the charity. The committee also reported the election of Sir Dyce Duckworth, M.D., LL.D., F.R.C.P., as a consulting physician, and that the requirements of the surgical work calling for the services of an additional surgeon, Mr. George Lenthal Cheatle, C.B., F.R.C.S., had been appointed to that office. Special mention was made of the presentation to the hospital of a Roentgen-ray apparatus from an anonymous donor (per Cav. Dr. F. Naumann), the cost of the installation of which was defrayed by Cav. E. Distin Maddick, and the committee of the popular ball; also the reflooring in mosaic, and tiling the walls, of the surgeons' consulting room by the Societa de Mosaicista Italiani, who crowned their work by placing in the room a handsome memorial tablet to the founder of the hospital (the late Com. J. Ortelli). In conclusion, the committee expressed their indebtedness to Mrs. Angiola Ortelli, the hon. lady superintendent; to the Very Rev. J. P. Bannin, the hon. chaplain; to Cav. Luciano de Rin, the hon. secretary; to the hon. medical staff and Dr. Pereira, the resident medical officer; and to the sister superior and the nursing staff.

In moving the adoption of the report, the CHAIRMAN, after eulogising the services of the secretary, Mr. Hornyik, touched on the various points mentioned, and emphasised the fact that though the moral and material progress of the institution had been most satisfactory, yet there was one dark point on the horizon—their financial difficulties. The deficit of close on £700 must be met somehow, and they greatly desired to open the remaining ten beds. In referring to the gift of £500 mentioned in the report, he said he did not know whether to be more pleased at the financial assistance afforded or at the motive which prompted it—friendship to Italy. Nothing could be more pleasing to an Italian Ambassador than to recognise that the Italian people had by their attitude shown once more that they could not forget the ties which bound them to this country. In addition to the £500 mentioned, another generous donor had come forward with a like sum; £200 had been collected from other sources; so that only £1,300 remained of the required amount. But the time was short, and he earnestly appealed both to the Italian colony in London and to their other friends to use their utmost endeavours to complete the total before the specified date.

Lord EDMUND TALBOT seconded the motion, which was agreed to.

The Rev. Father BANNIN announced, amid applause, that a further £300 had been received from a friend who desired to remain anonymous; and votes of thanks to the Press for their help, and to the Ambassador for presiding, having been adopted, the proceedings terminated.

### BRITISH LYING-IN HOSPITAL.

### SCHEME FOR A NURSES' HOME.

AT a special general meeting of the governors of the British Lying-in Hospital, Endell Street, on Tuesday, a resolution was moved by Mr. C. E. CHADWYCK HEALEY, K.C., empowering the expenditure by the board of £7,000 (£600 being for furniture) on a nurses' home. The object in view, said Mr. Healey, was to increase the accommodation. The governors would require to be satisfied not only of the necessity for this expenditure, but also that the accommodation to be provided should be the best possible. The subject had required very careful consideration, and it was now proposed by the board that the house in Betterton Street (the property of the hospital), which had been let at a rental of £84 a year, should be pulled down, and the new premises built on the site. Mr. Healy quoted the satistics of other London lying-in hospitals, showing the increase of patients in relation to the number of beds, the average number of patients per bed per annum being, at the British Hospital, the same as at Queen Charlotte's, viz., 24. In 1895 there were 202 patients, with 16 beds; and in 1901, 466 with only four more beds, while the number of nurses had also increased. It was thus evident that the increase was continuous and not sporadic, and it was plain that the utility of the hospital was growing. The figures for the last five years were: In 1897, 187 in-patients; in 1898, 269; in 1899, 331; in 1900, 354; and in 1901, 466. The Governors would appreciate the fact that with this increase of work there must be an increase of staff, but at the same time the accommodation had not increased at anything like the required rate. It was indeed by no means what it should be. The amount of work done in the twenty-four hours was much greater than it used to be a few years ago. It was proposed to provide in the new building for an increase of staff to the number of 10. At present there were four wards with 25 nurses; the alterations in Betterton Street would not only give better accommodation, but would also set free two rooms for wards. What, then, was it proposed to do? The plans before them on the table had been approved by the board; there would be 25 separate rooms (for some amount of privacy was required, even by a hospital nurse): the present dining-room, in which they had met, and another room behind, now occupied by four nurses (this gave some idea of the present accommodation) would be set free for wards, and there would be a proper room for the washing of the babies. These unhappy babies were now being washed in the labour room, which was not pleasant either for them or for their nurses, and there was a fear that the operation might sometimes have to be curtailed if the room were required. The new room would be large enough to wash 30 babies at a time, and to accommodate 30 babies' baskets, 15 washstands, and 15 chairs. The proposed alterations would very much improve the hospital, and he ventured to hope that the resolution would be carried without a dissentient voice. He hoped also that it would not be necessary to fall back upon the invested funds; the estimate was rather above than below what the actual cost would be, and had been founded on a wide basis.

Mr. T. A. NASH (trustee) seconded the resolution. There were one or two points which he wished to emphasise. First, as to the necessity for the proposed alterations, and secondly, whether the proposed scheme was the best that could be put before them. Mr. Chadwick Healey's figures had shown that during the past four years the number of in-patients had considerably more than doubled. To what was this increase due? Unquestionably, to the increased competency of the hospital. Poor women were realising the benefits of having the best medical skill and the most tender care during the hour of their greatest trial. The hospital was also an important training school; 130 ladies were trained every year, receiving certificates, and going out into the world to practise. They owed a duty to these ladies, who should be made as comfortable as possible during their term of training; their rooms should be healthy, airy, and commodious. At present they were not so. It was not right that ladies should have to turn out late at night in all sorts of weather, as three of the nurses did at present, being obliged to sleep at the house in Little Russell Street. After all, £7,000 was a mere mite compared with the sums which large hospitals not only asked for, but got. As one of the trustees, he hoped it would not be necessary to draw upon invested funds.

Dr. HANFIELD JONES supported the resolution. The increase in the number of patients, he said, was no flash in the pan, but proved the efficiency of the hospital. Again and again it had been his fate to come there in the small hours of the morning, to discuss with the matron as to where a newly-arrived patient was to be placed, and they had wondered what would happen if yet another came. The comparison with Queen Charlotte's Hospital was not quite fair in every way, because at the British Hospital only those cases were admitted that were absolutely deserving. It must be remembered that the work being done was not only a charity, but a service to the State.

The CHAIRMAN said this was the first time the British Hospital had come before the public asking for funds, and there was every reason to hope that these would be forthcoming. It did not seem very much to ask that ladies should help a hospital for poor married women, which aimed at giving a comfortable bed and skilled attention. Patients were kept longer than in other hospitals, viz., 15 days; it was formerly 18, and lately, owing to great pressure, it had been necessary to reduce the time as much as possible. They had always prided themselves on keeping the patients longer than other hospitals.

A GOVERNOR having asked how soon it was proposed to carry out the alterations, the CHAIRMAN replied that at the next board meeting orders would be given for demolition to begin.

There were present C. E. Farmer, Esq. (Chairman), C. E. Chadwyck Healey, Esq., K.C., the Rev. Canon Covington, J. A. Nash, Esq., Col. Farmer, A. B. Marten, Esq., and H. Fitzherbert Wright, Esq.

